# *In trans* variant calling reveals enrichment for compound heterozygous variants in genes involved in neuronal development and growth.

**DOI:** 10.1017/S0016672319000065

**Published:** 2019-06-13

**Authors:** Allison J. Cox, Fillan Grady, Gabriel Velez, Vinit B. Mahajan, Polly J. Ferguson, Andrew Kitchen, Benjamin W. Darbro, Alexander G. Bassuk

**Affiliations:** 1 Department of Pediatrics, The University of Iowa, Iowa City, IA, USA; 2 Interdisciplinary Graduate Program in Genetics, The University of Iowa, Iowa City, IA, USA; 3 Medical Scientist Training Program, University of Iowa, Iowa City, IA, USA; 4 Omics Laboratory, Department of Ophthalmology, Byers Eye Institute, Stanford University, Palo Alto, CA, USA; 5 Palo Alto Veterans Administration, Palo Alto, CA, USA; 6 Department of Anthropology, The University of Iowa, Iowa City, IA, USA

**Keywords:** bioinformatics, compound heterozygous, epilepsy, genetics

## Abstract

Compound heterozygotes occur when different variants at the same locus on both maternal and paternal chromosomes produce a recessive trait. Here we present the tool VarCount for the quantification of variants at the individual level. We used VarCount to characterize compound heterozygous coding variants in patients with epileptic encephalopathy and in the 1000 Genomes Project participants. The Epi4k data contains variants identified by whole exome sequencing in patients with either Lennox-Gastaut Syndrome (LGS) or infantile spasms (IS), as well as their parents. We queried the Epi4k dataset (264 trios) and the phased 1000 Genomes Project data (2504 participants) for recessive variants. To assess enrichment, transcript counts were compared between the Epi4k and 1000 Genomes Project participants using minor allele frequency (MAF) cutoffs of 0.5 and 1.0%, and including all ancestries or only probands of European ancestry. In the Epi4k participants, we found enrichment for rare, compound heterozygous variants in six genes, including three involved in neuronal growth and development – *PRTG* (p = 0.00086, 1% MAF, combined ancestries), *TNC* (p = 0.022, 1% MAF, combined ancestries) and *MACF1* (p = 0.0245, 0.5% MAF, EU ancestry). Due to the total number of transcripts considered in these analyses, the enrichment detected was not significant after correction for multiple testing and higher powered or prospective studies are necessary to validate the candidacy of these genes. However, *PRTG*, *TNC* and *MACF1* are potential novel recessive epilepsy genes and our results highlight that compound heterozygous variants should be considered in sporadic epilepsy.

## 1. Introduction

Using the premise that effective variants are in linkage disequilibrium (LD) with common polymorphisms and haplotypes, linkage and association studies have identified genes involved in the development of traits and pathologies. Upon their identification, the regions flanking associated markers are sequenced to find the linked, penetrant variant. However, rare variants are often not detectable using LD-based methods. This problem has been alleviated by recent advances in next-generation sequencing (NGS), and the detection of highly penetrant rare variants associated with disease has reduced the heritability gap for such diseases as autism, Crohn's disease and osteoporosis (Kosmicki *et al.*, 2016; Bomba, *et al.* 2017). Despite these advances, for most traits and complex disorders the underlying genes and variants remain elusive.

Recessive disorders are caused by mutations in both copies of a gene. The mutations may be homozygous, that is, identical or compound heterozygous. Compound heterozygous (CH) variants are two different variants in a gene on opposite alleles of a chromosome and it is speculated that CH mutations account for many recessive diseases (Li *et al.*, 2010; Sanjak *et al.*, 2017). Lack of detection of CH variants may explain a significant portion of missing heritability for all phenotypes (Li *et al.*, 2010; Zhong *et al.*, 2016; Sanjak *et al.*, 2017). Association studies using polymorphisms are LD-based and recent association studies using rare variants compare total variant burden between cases and controls to account for the contributions of multiple alleles at a locus to phenotype. Importantly, because LD-based studies require recessive variants to be on the same genetic background and total variant burden analyses are not allele specific, neither discerns between dominant and recessive models of inheritance.

Burden tests may account for compound heterozygosity if the variants are allocated to one of the two alleles for a gene, that is, phased. Relatively common variants may be phased assuming linkage to surrounding haplotypes; in families, rare variants are phased using parental genotypes. Once variants are phased, it may be determined if an individual's variants are on different chromosomes, and burden tests that aggregate using an indicator function (i.e. presence of qualifying variants) may assess enrichment for recessive variants.

Here we provide a publicly available tool, VarCount, that is user-friendly and effective for researchers seeking to quantify the presence or absence of a variant or variants in a gene at the individual level. VarCount is useful for the quantification of heterozygous, homozygous or CH variants per sample. We used VarCount to query the Epi4k (Epi4k Consortium *et al.*, 2013) dataset for rare homozygous and CH variants and found enrichment for rare, CH variants in six genes, including three involved in neuronal development or growth (*PRTG*, *TNC* and *MACF1*). The variants in the 1000 Genomes Project database are now phased (1000 Genomes Project Consortium *et al.*, 2010; 2012; 2015), and so genes may be queried for *in trans* combinations of variants. The Epi4k enrichment was identified in comparison to the 1000 Genomes Project participants combining all ancestries and considering only individuals of European ancestry.

## 2. Materials and methods

- Processing of Epi4k vcf files

The Epi4k data (Epi4k Consortium *et al.*, 2013) were accessed by permission via the Database of Genotypes and Phenotypes (dbGaP Study Accession, phs000653.v2.p1). Individual vcf files were combined using the CombineVariants function in GATK (McKenna *et al.*, 2010). The vcf files were then annotated with minor allele frequencies (MAFs) from EVS (Exome Variant Server, NHLBI GO Exome Sequencing Project [ESP], Seattle, WA [URL: http://evs.gs.washington.edu/EVS/]), 1000 Genomes Project and ExAC (Lek *et al.*, 2015), and with information regarding the effect of each variant using SNPSift/SNPEff (Cingolani *et al.*, 2012). The databases used for annotation were dbNSFP2.9 (for MAF and CADD score) and GRCh37.75 for protein effect prediction. SNPSift was used to remove any variants not inducing a protein-changing event (not ‘HIGH’ or ‘MODERATE’ impact) based on SNPEff annotation – this includes missense, nonsense, splice-site and insertion/deletion variants. Variants with quality flags and multiallelic variants, that is, those with more than two known nucleotide values, were also removed. Variants remaining after filtering were cross-referenced with the 1000 Genomes Project variants from the same MAF threshold to ensure that any variants removed from one dataset were removed from the other. The annotated vcf was used as input for VarCount. Ancestry for each exome was determined using LASER (Wang *et al.*, 2014) and this information was input to VarCount via the SampleInfo.txt file. Ancestry and phenotype information for each proband are described in Supplementary Table 1. In addition to the annotated vcf file, the parameters.txt and subjectinfo.txt (containing sex and ancestry information) were used as input. Within the parameters file, the following qualifications were selected: (1) counting at the transcript (rather than gene) level, (2) protein-changing effects, (3) MAF threshold of either 0.005 or 0.01, (4) all within-dataset and annotated (1000 Genomes Project, ExAC and EVS) MAFs, and (5) either CH or homozygous variants. Analyses were run separately for the two MAFs and using all Epi4k probands (264) and only those of European ancestry (207). Because the variants were not phased, VarCount was used to query the vcf file for individuals with two or more variants in each transcript. The output, a list of counts for each transcript was then used to query the parental vcf files for genotype information to determine which sets of variants composed *in trans* combinations of variants. Final counts were determined using parental genotype information. Custom python scripts were used to query for parental genotypes and to count true compound heterozygotes or homozygotes. *De novo* variants were excluded in the determination of true *in trans* variants.

- Processing of 1000 Genomes Project vcf files

**Fig. 1.**
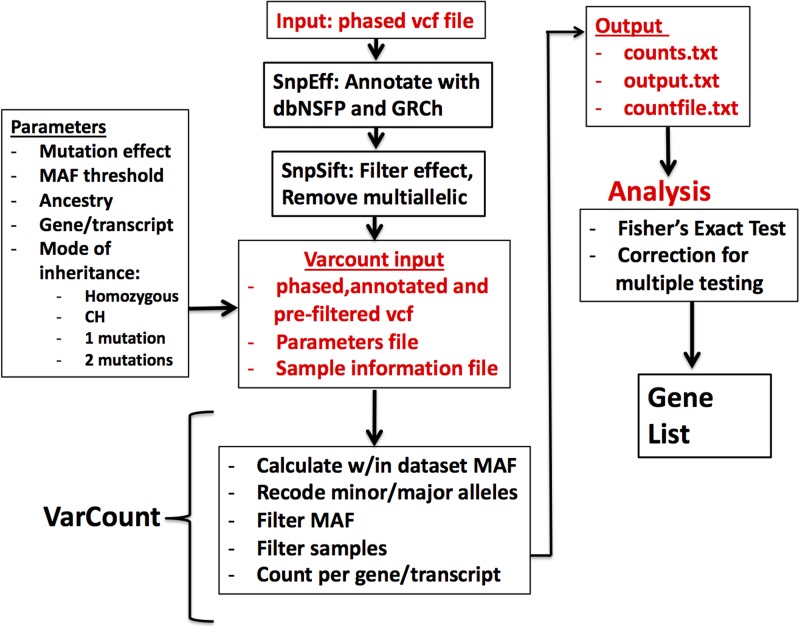
Flow diagram for the processing and analysis of variant lists. Vcf files are annotated and filtered using SNPSift/SNPEff. Final vcf along with parameter and sample information files are input to VarCount. The input files are processed to recode minor and major alleles when the MAF >0.5 and to count the number of individuals with variants qualifying based on information in the parameter file. The final output lists for every transcript or gene, the number of individuals with qualified variants in that locus (counts.text), which individuals have the variant(s) (countfile.txt), and which variants are harboured by each individual (output.txt).

Vcf files for the 2504 participants in the 1000 Genomes Project (1000 Genomes Project *et al.*, 2015) were downloaded by chromosome from the 1000 Genomes Project ftpsite. To reduce input file size, the genomic regions for the hg19 mRNA transcripts were downloaded via UCSC's Table Browser and used to remove noncoding regions from the vcf files. Including all exons from UCSC allowed for a more conservative analysis, given that the Epi4k data were sequenced using various exome captures, which are not inclusive of all possible exons. The variants were annotated and filtered via the same steps as the Epi4k vcf file. Multi-allelic variants were also removed prior to analysis by VarCount. A diagram showing the steps involved in processing and analysing the variant files is shown in (Figure 1).

Vcf files were queried for homozygous and CH variants using VarCount. Because the variants in the 1000 Genomes Project vcf files are phased, determining true compound heterozygotes is automatic using VarCount. In addition to the annotated vcf file, the parameters.txt and subjectinfo.txt (containing sex and ancestry information) were used as input. Within the parameters file, the same qualifications used in the Epi4k analysis were selected: analyses were run for each of the two MAFs (0.5 and 1.0%) and for all 1000 Genomes Project participants and using only those of European (EUR) ancestry. The final output from analyses was for each MAF cutoff and for each population, counts for every transcript in which at least one individual harboured recessive variants.

- Epi4k statistical analysis

Using R statistical software, a Fisher's exact test was used to detect transcripts with significant differences in the proportion of individuals with homozygous or CH variants between the Epi4k dataset and the 1000 Genomes Project dataset. Odds ratios and p-values were calculated using the number of individuals with and without qualifying variants. Analyses were performed using all ancestries, and for only individuals of European ancestry. Both Bonferroni and Benjamini-Hochberg adjustments were used to determine significance thresholds after correction for multiple testing. The number of tests was based on the number of transcripts with at least one individual in either the Epi4k or 1000 Genomes Project dataset with *in trans* coding variants with MAFs below the set threshold.

- Structural modelling of PRTG

The three-dimensional structure of Protogenin (PRTG) was modelled off the crystal structure of the human receptor protein tyrosine phosphatase sigma (PDB: 4PBX; 25.1% sequence identity) using MODELLER 9.14 (Webb and Sali, 2016). The resultant model superimposed with the template had an RSMD of 4.94 Å over 442 C*α* atoms. Charges and hydrogen atoms were added to the wild-type and mutant FGR models using PDB2PQR (Dolinsky *et al.*, 2004). Electrostatic potentials were calculated using APBS (Konecny *et al.*, 2012) as described previously (Moshfegh *et al.*, 2016; Cox *et al.*, 2017; Toral *et al.*, 2017). Protein and solvent dielectric constants were set to 2.0 and 78.0, respectively. All structural figures were generated by PyMOL (https://pymol.org/2/; Schrödinger, LLC).

## 3. Results

- Varcount: variant quantification at the individual level

Varcount is a free, open source tool useful for the quantification of heterozygous, homozygous or CH variants per sample. Input variants may be phased or unphased. All python scripts and supporting files may be downloaded from Github at https://github.com/GeneSleuth/VarCount. Supporting files include the ‘parameters.txt’ file where the user may select variant filters for variant effect, MAF and inheritance pattern (homozygous, CH, one variant or two variants), and sample filters based on information entered into the ‘SampleInfo file’. Input vcf files must be annotated with SNPSift/SNPEff using the dbNSFP and GRCh37/38 databases. A readme file with instructions is also provided. A flow diagram with the steps involved in processing of data is depicted in Figure 1.

- CH variants in Epi4k probands reveal novel epilepsy genes

We used VarCount to query the Epi4k dataset for rare homozygous and CH variants. The Epi4k data are whole exome data from 264 trios with a child affected by epileptic encephalopathy, either infantile spasms (IS) or Lennox-Gastaut Syndrome (LGS) (Epi4k Consortium et al., 2013). Counts were performed using individuals of all ancestries or just those of European ancestry (207/264). Individuals from the 1000 Genomes Project were used as controls. The individual counts and p-values for the analyses are listed in (Supplementary Tables 2–5). Including only rare variants (MAFs below 0.5 and 1.0%) determined enrichment for CH variants in six genes. For combined ancestries, the six genes are in order of significance: *OSBP2*, *PRTG*, *ABCC11*, *MACF1*, *STAB1* and *TNC*. *PRTG* and *TNC* were also highly ranked in the 1% MAF analysis, with one additional count for each transcript. Variants for all six genes are listed in Table 1. In our analysis of just individuals of European ancestry, *MACF1* was the most significantly enriched gene using a 0.5% MAF. The p-values indicated in Table 1 are for individual tests; there were no p-values significant after correction for multiple testing.

**Table 1.** Rare (<0.5 and 1.0% minor allele frequency) compound heterozygous variants in Epi4k participants.

Gene	Transcript	Sex	Phen^a^.	Anc^b^.	Chr:bp*	dbSNP ID	REF/ ALT	Exac ALL AF^c^	ExAC NFE AF	Peptide change	Epi4k # (1%)	1 kg # (1%)	p-value	Epi4k # (0.5%)	1 kg # (0.5%)	p-value
											EU (y/n)^d^: All (y/n)	EU (y/n): All (y/n)	EU:All	EU (y/n): All (y/n)	EU (y/n): All (s/n)	EU:All
*PRTG*	ENST000 00389286	F	IS	EU	15:56032666	rs373423650	T/C	9.94E-05	1.65E-04	E104G	3/204: 3/261	0/503: 0/2504	**0.0245: 0.00086**	2/205: 2/262	0/503: 0/2504	0.0847: **0.0091**
					15:56032666	rs185716584	C/G	0.003022	0.004315	E104D						
		M	LGS	EU	15:56032665	rs372777171	C/A	8.28E-06	1.50E-05	E575*						
					15:55965698	rs185716584	C/G	0.003022	0.004315	E104D						
		M	LGS	EU	15:56032665	rs35718474	G/A	0.003033	0.006701	P13S						
					15:56035093	rs148011047	T/G	0.002611	0.003608	K977T						
*OSBP2*	ENST000 00332585	M	IS	EU	15:55916703	rs200118898	C/G	1.09E-04	1.98E-04	S143W	2/205: 2/262	0/503: 0/2504	0.0847: 0.0091	2/205: 2/262	0/503: 0/2504	0.292: **0.0091**
					22:31091324	rs201298398	G/A	3.20E-04	5.19E-04	R262Q						
		M	LGS	E Asia	22:31137288	rs576508023	G/C	8.42E-06	0	G115R						
					22:31091239	NA	C/T	1.66E-05	0	R383W						
*ABCC11*	ENST000 00394747	F	IS	EU	22:31283452	rs529824818	C/T	8.24E-06	0	C543Y	2/205: 2/262	0/503: 2/2502	0.0847: 0.0478	2/205: 2/262	0/503: 1/2502	0.0847: 0.0255
					16:48242388	rs199839251	C/T	8.29E-06	1.51E-05	E273K						
		M	LGS	EU	16:48250159	NA	C/A	NA	NA	R889M						
					16:48226471	NA	C/A	NA	NA	G636V						
*TNC*	ENST000 00345230	M	IS	EU	16:48234362	rs200401362	C/T	8.24E-06	0	G861R	2/205: 3/261	0/503: 4/2500	0.0847: 0.022	1/206: 2/262	0/503: 2/2502	0.29: 0.0478
					9:117840315	rs117058692	C/G	7.77E-04	0.001248	G171R						
					9:117849499	rs143586851	C/T	2.23E-04	3.90E-04	A39T						
		M	IS	C/S Asia	9:117853183	rs149986851	C/A	2.06E-04	3.75E-04	G203V						
					9:117849402	rs139280264	G/A	8.51E-04	0.001247	R1066C						
		M	IS	EU	9:117835900	rs371055558	C/T	2.54E-05	3.07E-05	G576S						
					9:117848284	rs144032672	C/T	0.001336	0.002026	G210S						
*MACF1*	ENST000 00289893	M	IS	EU	9:117849382	NA	G/A	1.65E-05	3.00E-05	R4344Q	3/204: 3/261	1/502: 11/2493	0.0769: 0.142	3/204: 3/261	0/503: 6/2498	**0.0245**: 0.0478
					1:39900231	rs141949859	G/T	6.34E-04	1.12E-03	V3535F						
		M	IS	EU	1:39853797	rs145271544	G/T	NA	NA	A3264S						
					1:39852984	rs138819868	T/G	2.46E-03	3.85E-03	F5885L						
		M	LGS	EU	1:39951304	NA	A/G	8.28E-06	0	I1066V						
					1:39800136	NA	G/C	8.264	1.50E-05	W4967C						
*STAB1*	ENST000 00321725	M	IS	ME	1:39910474	rs145751447	G/A	9.17E-05	0	D1289N	0/207: 2/262	1/502: 6/2498	0.99: 0.173	0/207: 2/262	1/502: 2/2502	0.99: 0.0478
					3:52549439	rs147953260	G/A	0.001512	0.002524	R1872H						
		F	IS	C/S Asia	3:52554531	rs189303343	A/G	4.05E-05	3.66E-05	I590V						
					3:52540204	NA	C/T	NA	NA	R2351W						

^a^Phen = phenotype, ^b^Anc = ancestry, ^c^AF = allele frequency, ^d^y/n corresponds to yes/no counts of individuals with qualifying variants.

*bp (base pair position) in hg19/Build37.

Ancestries: EU = European, E Asia = East Asia, C/S Asia = Central/South Asia, ME = Middle East. Phenotypes: IS = Infantile spasms, LGS = Lennox-Gastaut syndrome.

p-values in bold are the most significant for the specific analysis.

**Fig. 2.**
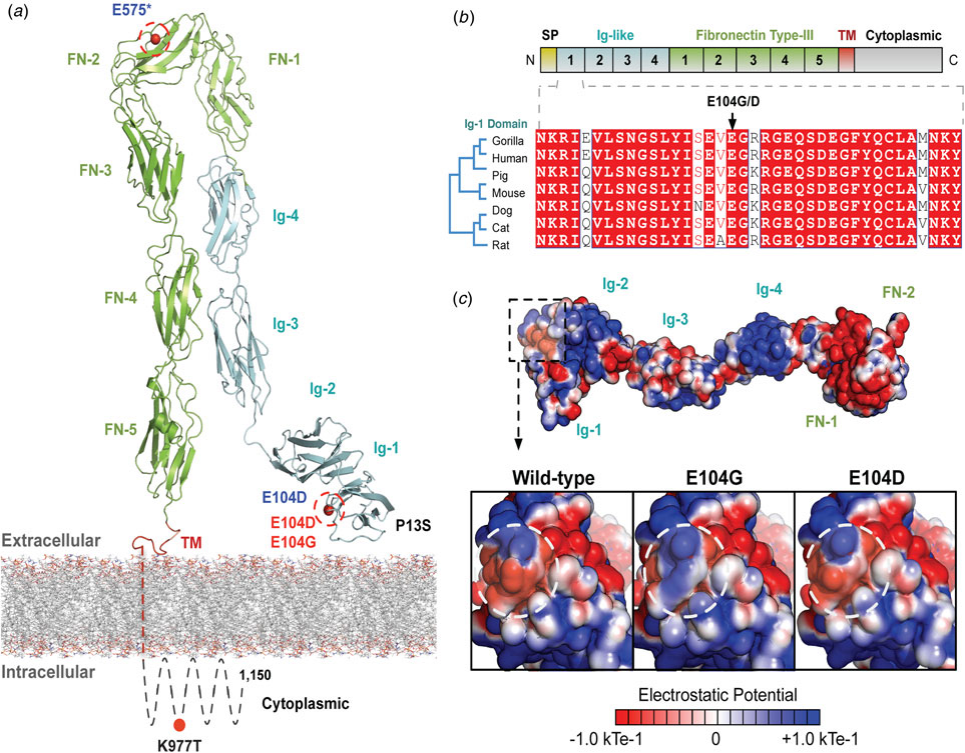
PRTG compound heterozygous mutations in Epi4k probands. (a) Theoretical model of the human PRTG structure spanning the plasma membrane indicating mutation locations in each child. The three pairs of *in trans* mutations, indicated in red, were found using a <1% MAF threshold. (b) Schematic representation of PRTG functional domains. Multiple sequence alignment of the PRTG Ig-1 domain. The E104 residue is 100% conserved across seven species. (c) Top: Electrostatic potential surface of PRTG calculated in APBS. Bottom: Close-up of the PRTG electrostatic potential surface at the site of mutation. The p.Glu104Gly mutation leads to a loss of negative charge, which may disrupt interactions with putative PRTG binding partners. The p.Glu104Asp mutation does not lead to a change in charge or electrostatic potential.

The variants for the three individuals with CH *PRTG* variants are depicted in Figure 2a. Because of the concentration of variants at position E104, we performed structural modelling to predict the pathogenicity of the PRTG variants. The p.Glu104Gly and p.Glu104Asp variants localize to the immunoglobulin (Ig)-like domain 1 (Figure 2a). Ig-like domains are responsible for mediating protein–protein and protein–peptide interactions. The p.Glu104Gly disrupts a negative charge in the Ig-like 1 domain. This loss of charge may disrupt interactions with putative PRTG-binding partners (Figure 2c).

The *de novo* variants identified by Epi4k Consortium and the Epilepsy Phenome/Genome Project (Epi4k Consortium *et al.*, 2013) in the nine probands with either *PRTG*, *TNC* or *MACF1* recessive variants are described in Table 2. For the three patients with CH *PRTG* variants, one patient harbours a *de novo* missense variant in *HSF2*, the second has a nonsense variant in *CELSR1*, and the third patient has two *de novo* variants – a missense in Fam102A and a 3´UTR variant in *USP42*. *De novo* mutations were only reported in one of the probands with *in trans TNC* variants – a missense variant in *DIP2C* and a splice donor change in *IFT172*. All three patients with CH variants in *MACF1* were reported to have *de novo* mutations. The first patient has a 5´ and 3´UTR *de novo* variant in *FAM19A2* and *GLRA2*, respectively, and the second patient also has a 3´UTR *de novo* change in the gene *LRRC8D* and a missense change in *SNX30*. One *de novo* variant was identified in the third proband in the gene *FAM227A.* Polyphen2 categories and CADD scores for each *de novo* variant as well as missense and loss-of-function constraint metric values for each gene (from ExAC) are also listed in Table 2. The z-score is a ratio of expected to identified missense variants in a particular gene, and pLI is a gene's probability of being loss-of-function intolerant. These constraint metrics are calculated using genomic data from controls without severe genetic diseases in the ExAC database (Lek *et al.*, 2015).

**Table 2.** *De novo* variants in Epi4k probands with compound heterozygous variants in *PRTG*, *TNC* or *MACF1*.

Gene	Missense z-score^a^	pLI^b^	CH variants	Sex	Phenotype	gene w/ *de novo*	Variant type	Polyphen-2^c^	CADD^d^	Missense z-score^a^	pLI^b^
*PRTG*	–0.92	0.02	E104G, E104D	F	IS	*HSF2*	Missense	P	24	1.77	0.93
*PRTG*			E104D, E575X	M	LGS	*CELSR1*	Stop-gained	NA	35	4.42	1.00
*PRTG*			P13S, K977T	M	LGS	*FAM102A*	Missense	D	25.1	1.80	0.43
						*USP42*	3´ UTR	NA		–0.69	1.00
*TNC*	–0.14	0.00	G861R, G171R, A39T	M	IS	*DIP2C*	Missense	B	23.6	5.82	1.00
						*IFT172*	Splice donor	NA	23.9	0.22	0.00
*TNC*			G203V, R1066C	M	IS	NA	–	–	–	–	–
*TNC*			G576S, G210S	M	IS	NA	–	–	–	–	–
*MACF1*	2.63	1.00	R4344Q, V3535F	M	IS	*FAM19A2*	5´ UTR	NA	NA	1.11	0.55
						*GLRA2*	3´ UTR	NA	NA	3.28	0.91
*MACF1*			A3264S, F5885L	M	IS	*LRRC8D*	3´ UTR	NA	NA	2.70	0.87
						*SNX30*	Missense	B	17.9	0.90	0.97
						*WDFY2*	Synonymous	NA	NA	1.15	0.00
*MACF1*			I1066V, W4967C	M	LGS	*FAM227A*	Missense	P	9.4	NA	NA

^a^z-score is a measure of tolerance to missense variants, based on ratio of expected to identified; ^b^pLI is the probability that a gene is intolerant to loss-of-function variants; ^c^Polyphen2 – prediction of a missense variant's impact on protein structure and function: B = benign, P = possibly damaging, D = damaging (Adzhubei *et al.*, 2010); ^d^CADD = phred-scaled score of Combined Annotation Dependent Depletion, a measure of the deleteriousness of a SNP or INDEL (Kircher *et al.*, 2014).

Phenotypes: IS = Infantile spasms, LGS = Lennox-Gastaut syndrome.

## 4. Discussion

Epileptic encephalopathies are a group of severe, early-onset seizure disorders with consistent EEG abnormalities that over time interfere with development and cause cognitive decline (Covanis, 2012). The Epi4k dataset contains exome sequences from 264 trios that include a proband with epileptic encephalopathy, either LGS or IS. LGS is characterized by frequent, mixed epileptic seizures that arise most frequently between the ages of 3 and 5 (Amrutkar & Riel-Romero, 2018). IS occurs during the first year of life and is cryptic in its presentation, with mild head bobbing and is often not detected until the seizures have caused significant neurological damage (Kossoff, 2010). IS often progress into LGS over time.

We developed a free and user-friendly tool, VarCount, to query vcf files for individuals harbouring variants that qualify according to user specification. To test its function, we used VarCount to quantify rare, CH variants in probands from the Epi4k trio dataset and found enrichment for variants in six genes including *PRTG*, *TNC* and *MACF1*. *PRTG* codes for protogenin, a member of the immunoglobulin superfamily that is involved in axis elongation and neuronal growth during early vertebrate development (Toyoda *et al.*, 2005; Vesque *et al.*, 2006). TNC and MACF1 are also directly involved in neuronal development and/or growth. TNC (Tenascin-C) is an extracellular matrix glycoprotein involved in axonal growth and guidance (Jakovcevski *et al.*, 2013). Seizures up-regulate *TNC* in the hippocampus, and in a pilocarpine epilepsy model up-regulation was shown to be mediated by TGF-*β* signalling (Mercado-Gomez *et al.*, 2014). MACF1 is a cytoskeletal crosslinking protein highly expressed in the brain and is crucial for neuron development and migration (Moffat *et al.*, 2017). *MACF1* variants are associated with the neurological pathologies Parkinson's disease, autism and schizophrenia (Moffat *et al.*, 2017). Recently, highly penetrant *de novo MACF1* mutations were identified in several patients with a newly characterized lissencephaly with a complex brain malformation (Dobyns *et al.*, 2018). This new phenotype highlights *MACF1* variants’ variable impact on disease pathogenesis. Given both the enrichment in Epi4k probands for CH variants in these genes as well as their known involvement in neuronal processes, we suggest that *PRTG*, *TNC* and *MACF1* are candidate recessive epilepsy genes.

The primary publication reporting analysis of the Epi4k trio dataset was a description of *de novo* mutations in the probands (Epi4k Consorium *et al.*, 2013). An analysis of CH variants was also reported, using a MAF cutoff of 0.15%, which is lower than the cutoff used in the work presented here. In this analysis, the parents were used as internal controls, and CH variants in 351 genes were identified, without genome-wide significance. The authors only listed five of the genes which are known to cause Mendelian disorders that include a seizure phenotype – *ASPM*, *CNTNAP2*, *GPR98*, *PCNT* and *POMGNT1*. In our analysis using the 1000 Genomes Project participants as controls, enrichment for CH variants was not detected in any of these genes. Using the number of individuals with *in trans* variants in a gene (transcript) as an indicator function required at least two probands to have qualifying variants in order to detect single-test significance, with complete absence of qualifying variants in controls. It is clear from the analyses using either internal controls or the 1000 Genomes Project as controls that a larger sample size is required to achieve genome-wide significance.

The *de novo* variants reported by the Epi4k Consortium and the Epilepsy Phenome/Genome Project (Epi4k Consortium *et al.*, 2013) in the nine probands with CH variants in *PRTG*, *TNC* or *MACF1* are described in Table 2. Of the 12 genes with *de novo* variants identified in the nine patients, three are implicated in neurological disease. *CELSR1* is a planar cell polarity gene in which mutations are known to cause neural tube defects including spina bifida (Robinson *et al.*, 2012). *De novo* deletions of *DIP2C* have been reported in two patients with cerebral palsy, one of whom also had ADHD, and the other had seizures in infancy (Zarrei *et al.*, 2018). In another report, deletions including *DIP2C* and/or *ZMYND11* were identified in several patients with developmental delay including three patients with seizures (DeScipio *et al.*, 2012). GLRA2 is a glycine receptor involved in neurodevelopment in which variants are implicated in autism (Pilorge *et al.*, 2016; Lin *et al.*, 2017), including a patient with comorbid epilepsy (Zhang *et al.*, 2017).

Of the *de novo* variants reported in these genes, the nonsense variant in *CELSR1* identified in one of the probands with *in trans PRTG* variants is the most likely to be pathogenic. However, regarding their involvement in neural tube defects, variants in *CELSR1* are thought to contribute to pathogenesis but not in a Mendelian fashion, as variants have been found to be inherited from unaffected parents or to be ineffective in functional assays (Robinson *et al.*, 2012; Allache *et al.*, 2012). The nonsense *CELSR1* variant in the patient reported here may contribute to epilepsy in the presence of a genetic modifier. The *de novo* missense mutation in *DIP2C* is predicted to be deleterious (CADD = 23.6) and has a low rate of benign missense variation based on constraint metrics (z = 5.82). The *de novo* variant in *GLRA2* is in the 3´UTR so it is difficult to predict its impact on gene function and subsequent pathogenicity.

The CH variants in *PRTG*, *TNC* and *MACF1* are similarly variable in predicted pathogenicity, with CADD scores ranging from between less than one to 38. *PRTG* and *TNC* both have constraint metrics indicative of a high tolerance to both missense and loss-of-function variants, while *MACF1* is moderately intolerant of missense variants (z = 2.63) and extremely intolerant of loss-of-function variants (pLI = 1.0). Interestingly, aside from the 3´UTR variant in *GLRA2*, none of the *de novo* variants in the Epi4k participants with *MACF1* CH variants are in genes associated with neurological disease or predicted with confidence to have a negative impact on gene function. This, in addition to *MACF1*’s intolerance to missense or nonsense variants, is supportive of the pathogenicity of the biallelic variants in the gene.

In summary, we present a free tool VarCount for the quantification of qualifying variants as an indicator function per individual in the analysis of variant lists (vcf files). We used VarCount to assess enrichment of rare, coding, CH variants in a cohort of 264 epilepsy probands and found enrichment in three genes involved in neurodevelopmental processes – *PRTG*, *TNC* and *MACF1*. A missense change at the E104 residue of PRTG was identified three times in two different probands. Significance was not maintained after correction for multiple testing, and larger cohorts or candidate gene studies using a different sample set are necessary to validate this enrichment. In the context of the *de novo* mutations also present in these patients, experimentation is necessary in order to delineate if the CH or *de novo* variants, or both, are pathogenic in the development of epileptic encephalopathy. *PRTG*, *TNC* and *MACF1* are candidate recessive epilepsy genes and our work highlights that inheritance of CH variants should not be excluded from gene discovery or diagnostic analyses of patients with epilepsy.

## Supplementary material

For supplementary material accompanying this paper visit https://doi.org/10.1017/S0016672319000065. click here to view supplementary material

## References

[ref1] 1000 Genomes Project Consortium, Abecasis GR, Altshuler D, Auton A, Brooks LD, Durbin RM, Gibbs RA, Hurles ME and McVean GA (2010). A map of human genome variation from population-scale sequencing. Nature 467(7319), 1061–1073. 20981092 10.1038/nature09534 PMC3042601

[ref2] 1000 Genomes Project Consortium, Abecasis GR, Auton A, Brooks LD, DePristo MA, Durbin RM, Handsaker RE, Kang HM, Marth GT and McVean GA (2012). An integrated map of genetic variation from 1,092 human genomes. Nature 491(7422), 56–65. 23128226 10.1038/nature11632 PMC3498066

[ref3] 1000 Genomes Project Consortium, Auton A, Brooks LD, Durbin RM, Garrison EP, Kang HM, Korbel JO, Marchini JL, McCarthy S, McVean GA and Abecasis GR (2015). A global reference for human genetic variation. Nature 526(7571), 68–74. 26432245 10.1038/nature15393 PMC4750478

[ref4] Adzhubei IA, Schmidt S, Peshkin L, Ramensky VE, Gerasimova A, Bork P, Kondrashov AS and Sunyaev SR (2010). A method and server for predicting damaging missense mutations. Nature Methods 7(4), 248–249. 20354512 10.1038/nmeth0410-248 PMC2855889

[ref5] Allache R, De Marco P, Merello E, Capra V and Kibar Z (2012). Role of the planar cell polarity gene *CELSR*1 in neural tube defects and caudal agenesis. Birth Defects Research A. Clinical and Molecular Teratology 94(3), 176–181. 22371354 10.1002/bdra.23002

[ref6] Amrutkar C and Riel-Romero RM (2018). Lennox Gastaut syndrome. Treasure Island, FL: StatPearls.

[ref7] Bomba L, Walter K and Soranzo N (2017). The impact of rare and low-frequency genetic variants in common disease. Genome Biology 18(1), 77. 28449691 10.1186/s13059-017-1212-4 PMC5408830

[ref8] Cingolani P, Platts A, Wang le L, Coon M, Nguyen T, Wang L, Land SJ, Lu X and Ruden DM (2012). A program for annotating and predicting the effects of single nucleotide polymorphisms, SNPEff: SNPs in the genome of *Drosophila melanogaster* strain w1118; iso-2; iso-3. Fly *(*Austin*)* 6(2), 80–92. 22728672 10.4161/fly.19695 PMC3679285

[ref9] Covanis A (2012). Epileptic encephalopathies (including severe epilepsy syndromes). *Epilepsia* 53 Suppl. 4, 114–126. 10.1111/j.1528-1167.2012.03621.x 22946729

[ref10] Cox AJ, Darbro BW, Laxer RM, Velez G, Bing X, Finer AL, Erives A, Mahajan VB, Bassuk AG and Ferguson PJ (2017). Recessive coding and regulatory mutations in FBLIM1 underlie the pathogenesis of chronic recurrent multifocal osteomyelitis (CRMO). PLoS One 12(3), e0169687. 28301468 10.1371/journal.pone.0169687 PMC5354242

[ref11] Dobyns WB, Aldinger KA, Ishak GE, Mirzaa GM, Timms AE, Grout ME, Dremmen MHG, Schot R, Vandervore L, van Slegtenhorst MA, Wilke M, Kasteleijn E, Lee AS, Barry BJ, Chao KR, Szczałuba K, Kobori J, Hanson-Kahn A, Bernstein JA, Carr L, D'Arco F, Miyana K, Okazaki T, Saito Y, Sasaki M, Das S, Wheeler MM, Bamshad MJ, Nickerson DA, University of Washington Center for Mendelian Genomics, Center for Mendelian Genomics at the Broad Institute of MIT and Harvard, Engle EC, Verheijen FW, Doherty D and Mancini GMS (2018). MACF1 mutations encoding highly conserved zinc-binding residues of the GAR domain cause defects in neuronal migration and axon guidance. American Journal of Human Genetics 103(6), 1009–102. 30471716 10.1016/j.ajhg.2018.10.019 PMC6288423

[ref12] DeScipio C, Conlin L, Rosenfeld J, Tepperberg J, Pasion R, Patel A, McDonald MT, Aradhya S, Ho D, Goldstein J, McGuire M, Mulchandani S, Medne L, Rupps R, Serrano AH, Thorland EC, Tsai AC, Hilhorst-Hofstee Y, Ruivenkamp CA, Van Esch H, Addor MC, Martinet D, Mason TB, Clark D, Spinner NB and Krantz ID (2012). Subtelomeric deletion of chromosome 10p15.3: clinical findings and molecular cytogenetic characterization. American Journal of Medical Genetics Part A 158A(9), 2152–2161. 22847950 10.1002/ajmg.a.35574 PMC3429713

[ref13] Dolinsky TJ, Nielsen JE, McCammon JA and Baker NA (2004). PDB2PQR: an automated pipeline for the setup of Poisson-Boltzmann electrostatics calculations. *Nucleic Acids Research* 32(Web Server issue), W665–W667. 10.1093/nar/gkh381 PMC441519 15215472

[ref14] Epi4k Consortium, Epilepsy Phenome/Genome Project, Allen AS, Berkovic SF, Cossette P, Delanty N, Dlugos D, Eichler EE, Epstein MP, Glauser T, Goldstein DB, Han Y, Heinzen EL, Hitomi Y, Howell KB, Johnson MR, Kuzniecky R, Lowenstein DH, Lu YF, Madou MR, Marson AG, Mefford HC, Esmaeeli Nieh S, O'Brien TJ, Ottman R, Petrovski S, Poduri A, Ruzzo EK, Scheffer IE, Sherr EH, Yuskaitis CJ, Abou-Khalil B, Alldredge BK, Bautista JF, Berkovic SF, Boro A, Cascino GD, Consalvo D, Crumrine P, Devinsky O, Dlugos D, Epstein MP, Fiol M, Fountain NB, French J, Friedman D, Geller EB, Glauser T, Glynn S, Haut SR, Hayward J, Helmers SL, Joshi S, Kanner A, Kirsch HE, Knowlton RC, Kossoff EH, Kuperman R, Kuzniecky R, Lowenstein DH, McGuire SM, Motika PV, Novotny EJ, Ottman R, Paolicchi JM, Parent JM, Park K, Poduri A, Scheffer IE, Shellhaas RA, Sherr EH, Shih JJ, Singh R, Sirven J, Smith MC, Sullivan J, Lin Thio L, Venkat A, Vining EP, Von Allmen GK, Weisenberg JL, Widdess-Walsh P and Winawer MR (2013). *De novo* mutations in epileptic encephalopathies. Nature 501(7466), 217–221. 23934111 10.1038/nature12439 PMC3773011

[ref15] Jakovcevski I, Miljkovic D, Schachner M and Andjus PR (2013). Tenascins and inflammation in disorders of the nervous system. Amino Acids 44(4), 1115–1127. 23269478 10.1007/s00726-012-1446-0

[ref16] Kircher M, Witten DM, Jain P, O'Roak BJ, Cooper GM and Shendure J (2014). A general framework for estimating the relative pathogenicity of human genetic variants. Nature Genetics 46(3), 310–315. 24487276 10.1038/ng.2892 PMC3992975

[ref17] Konecny R, Baker NA and McCammon JA (2012). iAPBS: a programming interface to Adaptive Poisson-Boltzmann Solver (APBS). Computational Science and Discovery 5(1), pii: 015005. 22905037 10.1088/1749-4699/5/1/015005 PMC3419494

[ref18] Kosmicki JA, Churchhouse CL, Rivas MA and Neale BM (2016). Discovery of rare variants for complex phenotypes. Human Genetics 135(6), 625–634. 27221085 10.1007/s00439-016-1679-1 PMC6693675

[ref19] Kossoff EH (2010). Infantile spasms. Neurologist 16(2), 69–75. 20220440 10.1097/NRL.0b013e3181d1416c

[ref20] Lek M, Karczewski K, Minikel E, Samocha K, Banks E, Fennell T, O'Donnell-Luria A, Ware J, Hill A, Cummings B, Tukiainen T, Birnbaum D, Kosmicki J, Duncan L, Estrada K, Zhao F, Zou J, Pierce-Hoffman E, Cooper D, DePristo M, Do R, Flannick J, Fromer M, Gauthier L, Goldstein J, Gupta N, Howrigan D, Kiezun A, Kurki M, Moonshine AL, Natarajan P, Orozco L, Peloso G, Poplin R, Rivas M, Ruano-Rubio V, Ruderfer D, Shakir K, Stenson P, Stevens C, Thomas B, Tiao G, Tusie-Luna M, Weisburd B, Won H, Yu D, Altshuler D, Ardissino D, Boehnke M, Danesh J, Roberto E, Florez J, Gabriel S, Getz G, Hultman C, Kathiresan S, Laakso M, McCarroll S, McCarthy M, McGovern D, McPherson R, Neale B, Palotie A, Purcell S, Saleheen D, Scharf J, Sklar P, Patrick S, Tuomilehto J, Watkins H, Wilson J, Daly M and MacArthur D. (2016). Analysis of protein-coding genetic variation in 60,706 humans. Nature 536(7616), 285–291. 27535533 10.1038/nature19057 PMC5018207

[ref21] Li Y, Vinckenbosch N, Tian G, Huerta-Sanchez E, Jiang T, Jiang H, Albrechtsen A, Andersen G, Cao H, Korneliussen T, Grarup N, Guo Y, Hellman I, Jin X, Li Q, Liu J, Liu X, Sparsø T, Tang M, Wu H, Wu R, Yu C, Zheng H, Astrup A, Bolund L, Holmkvist J, Jørgensen T, Kristiansen K, Schmitz O, Schwartz TW, Zhang X, Li R, Yang H, Wang J, Hansen T, Pedersen O, Nielsen R and Wang J (2010). Resequencing of 200 human exomes identifies an excess of low-frequency non-synonymous coding variants. Nature Genetics 42(11), 969–972. 20890277 10.1038/ng.680

[ref22] Lin MS, Xiong WC, Li SJ, Gong Z, Cao X, Kuang XJ, Zhang Y, Gao TM, Mechawar N, Liu C and Zhu XH (2017). alpha2-glycine receptors modulate adult hippocampal neurogenesis and spatial memory. Develeopmental Neurobiology 77(12), 1430–1441. 10.1002/dneu.22549 29057625

[ref23] McKenna A, Hanna M, Banks E, Sivachenko A, Cibulskis K, Kernytsky A, Garimella K, Altshuler D, Gabriel S, Daly M and DePristo MA (2010). The Genome Analysis Toolkit: a MapReduce framework for analyzing next-generation DNA sequencing data. Genome Research 20(9), 1297–1303. 20644199 10.1101/gr.107524.110 PMC2928508

[ref24] Mercado-Gomez O, Mercado-Gómez O, Landgrave-Gómez J, Arriaga-Avila V, Nebreda-Corona A and Guevara-Guzmán R (2014). Role of TGF-beta signaling pathway on Tenascin C protein upregulation in a pilocarpine seizure model. Epilepsy Research 108(10), 1694–1704. 25445237 10.1016/j.eplepsyres.2014.09.019

[ref25] Moffat JJ, Ka M, Jung EM, Smith AL and Kim WY (2017). The role of MACF1 in nervous system development and maintenance. Seminars in Cell and Developmental Biology 69, 9–17. 28579452 10.1016/j.semcdb.2017.05.020 PMC5583038

[ref26] Moshfegh Y, Velez G, Li Y, Bassuk AG, Mahajan VB and Tsang SH (2016). BESTROPHIN1 mutations cause defective chloride conductance in patient stem cell-derived RPE. Human Molecular Genetics 25(13), 2672–2680. 27193166 10.1093/hmg/ddw126 PMC5181636

[ref27] Pilorge M, Fassier C, Le Corronc H, Potey A, Bai J, De Gois S, Delaby E, Assouline B, Guinchat V, Devillard F, Delorme R, Nygren G, Råstam M, Meier JC, Otani S, Cheval H, James VM, Topf M, Dear TN, Gillberg C, Leboyer M, Giros B, Gautron S, Hazan J, Harvey RJ, Legendre P and Betancur C (2016). Genetic and functional analyses demonstrate a role for abnormal glycinergic signaling in autism. Molecular Psychiatry 21(7), 936–945. 26370147 10.1038/mp.2015.139 PMC5382231

[ref28] Robinson A, Escuin S, Doudney K, Vekemans M, Stevenson RE, Greene ND, Copp AJ and Stanier P (2012). Mutations in the planar cell polarity genes CELSR1 and SCRIB are associated with the severe neural tube defect craniorachischisis. Human Mutation 33(2), 440–447. 22095531 10.1002/humu.21662 PMC4772123

[ref29] Sanjak JS, Long AD and Thornton KR (2017). A model of compound heterozygous, loss-of-function alleles is broadly consistent with observations from complex-disease GWAS datasets. PLoS Genetics 13(1), e1006573. 28103232 10.1371/journal.pgen.1006573 PMC5289629

[ref30] Toral MA, Velez G, Boudreault K, Schaefer KA, Xu Y, Saffra N, Bassuk AG, Tsang SH and Mahajan VB (2017). Structural modeling of a novel SLC38A8 mutation that causes foveal hypoplasia. Molecular Genetics and Genomic Medicine 5(3), 202–209. 28546991 10.1002/mgg3.266 PMC5441399

[ref31] Toyoda R, Nakamura H and Watanabe Y (2005). Identification of protogenin, a novel immunoglobulin superfamily gene expressed during early chick embryogenesis. Gene Expression Patterns 5(6), 778–785. 15922677 10.1016/j.modgep.2005.04.001

[ref32] Vesque C, Anselme I, Couve E, Charnay P and Schneider-Maunoury S (2006). Cloning of vertebrate Protogenin (Prtg) and comparative expression analysis during axis elongation. Developmental Dynamics 235(10), 2836–2844. 16881056 10.1002/dvdy.20898

[ref33] Wang C, Zhan X, Bragg-Gresham J, Kang HM, Stambolian D, Chew EY, Branham KE, Heckenlively J, Fulton R, Wilson RK, Mardis ER, Lin X, Swaroop A, Zollner S and Abecasis GR (2014). Ancestry estimation and control of population stratification for sequence-based association studies. Nature Genetics 46(4), 409–415. 24633160 10.1038/ng.2924 PMC4084909

[ref34] Webb B and Sali A (2016). Comparative protein structure modeling using MODELLER. Current Protocols in Protein Science 86, 2.9.1–2.9.37. 10.1002/cpps.20 27801516

[ref35] Zarrei M, Fehlings DL, Mawjee K, Switzer L, Thiruvahindrapuram B, Walker S, Merico D, Casallo G, Uddin M, MacDonald JR, Gazzellone MJ, Higginbotham EJ, Campbell C, deVeber G, Frid P, Gorter JW, Hunt C, Kawamura A, Kim M, McCormick A, Mesterman R, Samdup D, Marshall CR, Stavropoulos DJ, Wintle RF and Scherer SW (2018). *De novo* and rare inherited copy-number variations in the hemiplegic form of cerebral palsy. Genetics in Medicine 20(2), 172–180. 28771244 10.1038/gim.2017.83 PMC5846809

[ref36] Zhang Y, Ho TNT, Harvey RJ, Lynch JW and Keramidas A (2017). Structure-function analysis of the GlyR alpha2 subunit autism mutation p.R323L reveals a gain-of-function. Frontiers in Molecular Neuroscience 10, 158. 28588452 10.3389/fnmol.2017.00158 PMC5440463

[ref37] Zhong K, Karssen LC, Kayser M and Liu F (2016). CollapsABEL: an R library for detecting compound heterozygote alleles in genome-wide association studies. BMC Bioinformatics 17, 156. 27059780 10.1186/s12859-016-1006-9 PMC4826552

