# Supplementary material for: In trans variant calling reveals enrichment for compound heterozygous variants in genes involved in neuronal development and growth
Source: Genet Res (Camb). 2019 Jun 13;101:e8. doi: 10.1017/S0016672319000065 (PMC7045018; doi:10.1017/S0016672319000065)
Supplement: Supplementary file 1 [file S0016672319000065sup001.doc]

**Supplementary Table 1**: Ancestry and Phenotype Information for Epi4k probands

| **proband ID** | **TRACE^a^ ancestry*** | **Reported Ancestry** | **IS/LGS^b^** | **Sex** | **proband ID** | **TRACE ancestry** | **Reported Ancestry** | **IS/LGS** | **Sex** |
| --- | --- | --- | --- | --- | --- | --- | --- | --- | --- |
| isnd21451b1 | C/S Asia | Asian | IS | F | isnd35150fl1 | EU | White | IS | M |
| isnd21751e1 | EU | White | IS | M | isnd35151fo1 | EU | White | IS | M |
| isnd22993f1 | EU | White | IS | M | isnd35197fn1 | EU | White | IS | F |
| isnd23231g1 | EU | Other | IS | M | isnd35351fz1 | America | White | IS | M |
| isnd23465h1 | EU | White | IS | M | isnd35575et1 | EU | White | IS | F |
| isnd24005j1 | EU | White | IS | M | isnd35845ek1 | ME | Other | IS | M |
| isnd24104l1 | C/S Asia | Asian | IS | M | isnd35907fs1 | Africa | Other | IS | M |
| isnd24188n1 | EU | White | IS | F | isnd35929gc1 | EU | White | IS | M |
| isnd24217o1 | EU | White | IS | M | isnd35951fu1 | EU | White | IS | M |
| isnd24290af1 | ME | Hispanic | IS | M | isnd36066fd1 | EU | White | IS | F |
| isnd24346p1 | ME | White | IS | M | isnd36158fv1 | EU | White | IS | F |
| isnd24470t1 | EU | White | IS | M | isnd36206fw1 | C/S Asia | White | IS | F |
| isnd24539d1 | America | Hispanic | IS | M | isnd36211dg1 | EU | White | IS | F |
| isnd24704r1 | EU | White | IS | F | isnd36367fx1 | C/S Asia | Asian | IS | F |
| isnd24782s1 | America | Hispanic | IS | M | isnd36387fy1 | EU | White | IS | F |
| isnd25070u1 | EU | Other | IS | F | isnd36561ga1 | EU | White | IS | M |
| isnd25181w1 | EU | White | IS | M | isnd36610ge1 | EU | White | IS | M |
| isnd25582x1 | EU | White | IS | F | lgsnd22752gg1 | EU | Other | LGS | M |
| isnd25606v1 | EU | White | IS | F | lgsnd23319gi1 | America | Hispanic | LGS | M |
| isnd25793ac1 | EU | White | IS | M | lgsnd23543gr1 | EU | White | LGS | M |
| isnd25839z1 | Africa | African American | IS | M | lgsnd23813gv1 | EU | White | LGS | M |
| isnd26087ad1 | America | Hispanic | IS | F | lgsnd23828gl1 | EU | White | LGS | M |
| isnd26900ah1 | EU | White | IS | F | lgsnd24053gj1 | EU | White | LGS | F |
| isnd26970ai1 | EU | White | IS | F | lgsnd24065go1 | EU | White | LGS | M |
| isnd26974aj1 | EU | White | IS | F | lgsnd24070gk1 | EU | White | LGS | M |
| isnd27062aa1 | EU | White | IS | F | lgsnd24191jw1 | EU | White | LGS | M |
| isnd27253al1 | EU | White | IS | F | lgsnd24447gm1 | EU | White | LGS | F |
| isnd27474am1 | EU | White | IS | F | lgsnd24471gn1 | EU | White | LGS | M |
| isnd27521bi1 | EU | Other | IS | M | lgsnd24646gp1 | EU | White | LGS | F |
| isnd27732an1 | Africa | Other | IS | M | lgsnd24762gq1 | C/S Asia | White | LGS | F |
| isnd27841ay1 | Africa | African American | IS | M | lgsnd25442gs1 | EU | White | LGS | F |
| isnd27935ar1 | EU | White | IS | M | lgsnd25544gt1 | EU | White | LGS | F |
| isnd27949ag1 | EU | White | IS | M | lgsnd25992hc1 | EU | Other | LGS | M |
| isnd28478au1 | EU | White | IS | F | lgsnd26319gy1 | EU | White | LGS | F |
| isnd28661av1 | EU | White | IS | M | lgsnd27109hd1 | EU | White | LGS | F |
| isnd28699ax1 | EU | White | IS | M | lgsnd27155he1 | EU | White | LGS | F |
| isnd28895fp1 | EU | White | IS | M | lgsnd27345hh1 | EU | White | LGS | F |
| isnd28982bc1 | EU | White | IS | M | lgsnd27497hi1 | EU | White | LGS | M |
| isnd29057be1 | EU* | White | IS | F | lgsnd27543hn1 | E Asia | Asian | LGS | F |
| isnd29126bj1 | EU | White | IS | M | lgsnd27594hj1 | EU | Other | LGS | M |
| isnd29199bk1 | C/S Asia | White | IS | M | lgsnd27637hk1 | EU | White | LGS | M |
| isnd29258bl3 | EU | White | IS | M | lgsnd27682hl1 | EU | White | LGS | M |
| isnd29267bm1 | EU | Other | IS | M | lgsnd27753ha1 | EU | White | LGS | M |
| isnd29292ca1 | EU/CS | White | IS | F | lgsnd27952in1 | EU | White | LGS | F |
| isnd29305co1 | EU | White | IS | M | lgsnd28027hw1 | EU | White | LGS | M |
| isnd29319bo1 | EU | White | IS | F | lgsnd28181hp1 | EU | White | LGS | F |
| isnd29352br1 | EU | White | IS | F | lgsnd28245hq1 | EU | White | LGS | M |
| isnd29366bs1 | EU | White | IS | F | lgsnd28402ip1 | EU | White | LGS | M |
| isnd29377bt1 | ME  C/S Asia | Other | IS | F | lgsnd28432hu1 | EU | White | LGS | M |

^a^Ancestry determined by LASER/TRACE software, ^b^IS = Infantile Spasms, LGS = Lennox Gastaut Syndrome

*Ancestries: C/S Asia = Central/South Asia, E Asia = East Asia, EU = European, ME = ME

**Supplementary Table 1 Continued: Ancestry and Phenotype Information for Epi4k** probands

| **proband ID** | **TRACE^a^ ancestry*** | **Reported Ancestry** | **IS/LGS^b^** | **Sex** | **proband ID** | **TRACE ancestry** | **Reported Ancestry** | **IS/LGS** | **Sex** |
| --- | --- | --- | --- | --- | --- | --- | --- | --- | --- |
| isnd29378bu1 | EU | Other | IS | M | lgsnd28509hr1 | EU | White | LGS | M |
| isnd29383bv1 | C/S Asia | White | IS | M | lgsnd28633ht1 | EU | White | LGS | M |
| isnd29429es1 | EU | White | IS | M | lgsnd28840hf1 | EU | White | LGS | M |
| isnd29514bw1 | EU | White | IS | F | lgsnd28866hs1 | EU | White | LGS | F |
| isnd29556k1 | EU/CS Asia | White | IS | F | lgsnd28881hv1 | C/S Asia | Other | LGS | F |
| isnd29711ao1 | EU | White | IS | F | lgsnd28949hx1 | EU | White | LGS | M |
| isnd29810bq1 | EU | White | IS | F | lgsnd29055hz1 | EU | Other | LGS | M |
| isnd29844az1 | EU | White | IS | F | lgsnd29058ia1 | EU | White | LGS | F |
| isnd29865by1 | EU | White | IS | M | lgsnd29125iz1 | E Asia | Asian | LGS | M |
| isnd29900bg1 | EU | White | IS | F | lgsnd29146ic1 | EU | White | LGS | M |
| isnd30071cf1 | ME | Other | IS | M | lgsnd29196ig1 | EU | White | LGS | F |
| isnd30086ak1 | EU | White | IS | F | lgsnd29374gh1 | EU | White | LGS | M |
| isnd30090dk1 | EU | Other | IS | M | lgsnd29394ie1 | EU | Other | LGS | F |
| isnd30279ci1 | EU | White | IS | M | lgsnd29446if1 | ME | White | LGS | M |
| isnd30280cj1 | EU | White | IS | F | lgsnd29528ih1 | EU | White | LGS | M |
| isnd30302ck1 | EU | White | IS | M | lgsnd29554gw1 | America | Hispanic | LGS | M |
| isnd30373dm1 | EU | White | IS | M | lgsnd29789ii1 | EU | White | LGS | M |
| isnd30377cm1 | EU | White | IS | M | lgsnd29838ij1 | EU | Hispanic | LGS | F |
| isnd30384cn1 | EU | White | IS | M | lgsnd29864it1 | EU | White | LGS | M |
| isnd30431cp1 | C/S Asia | White | IS | F | lgsnd29904ik1 | Africa | Other | LGS | M |
| isnd30439cv1 | EU | White | IS | M | lgsnd29958il1 | EU | White | LGS | M |
| isnd30441cr1 | C/S Asia | White | IS | M | lgsnd30052je1 | EU | White | LGS | M |
| isnd30474ch1 | EU/CS | Other | IS | F | lgsnd30133iq1 | EU | White | LGS | M |
| isnd30482dy1 | EU | White | IS | F | lgsnd30216iy1 | EU | White | LGS | F |
| isnd30485cs1 | EU | White | IS | M | lgsnd30241ir1 | EU | White | LGS | M |
| isnd30552ct1 | EU | White | IS | F | lgsnd30378ix1 | EU | White | LGS | M |
| isnd30575cz1 | EU | White | IS | F | lgsnd30383iv1 | ME | White | LGS | M |
| isnd30610cg1 | EU | White | IS | M | lgsnd30631iw1 | EU | White | LGS | M |
| isnd30629gb1 | EU | Other | IS | F | lgsnd30729js1 | EU | White | LGS | F |
| isnd30679cu1 | EU | White | IS | M | lgsnd30798ja1 | EU | White | LGS | F |
| isnd30831dl1 | EU | White | IS | M | lgsnd30864jf1 | EU | White | LGS | M |
| isnd30880cx1 | EU | White | IS | M | lgsnd30965jg1 | E Asia | Asian | LGS | M |
| isnd30915as1 | EU | White | IS | M | lgsnd31059jh1 | EU | White | LGS | M |
| isnd31115da1 | EU | White | IS | F | lgsnd31063ji1 | EU | White | LGS | F |
| isnd31120cq1 | EU | Other | IS | M | lgsnd31153jj1 | EU | White | LGS | F |
| isnd31134ba1 | EU | White | IS | M | lgsnd31159jd1 | EU | White | LGS | F |
| isnd31192db1 | EU | White | IS | M | lgsnd31244jk1 | Africa | Other | LGS | M |
| isnd31228dc1 | EU | White | IS | M | lgsnd31529id1 | C/S Asia | Other | LGS | M |
| isnd31241dr1 | EU | White | IS | F | lgsnd31533jo1 | EU | Other | LGS | M |
| isnd31305dd1 | E Asia | Asian | IS | F | lgsnd31574jm1 | EU | White | LGS | M |
| isnd31308de1 | C/S Asia | Asian | IS | F | lgsnd31650jv1 | EU | White | LGS | M |
| isnd31364df1 | EU | White | IS | M | lgsnd31664jn1 | EU | White | LGS | M |
| isnd31602ds1 | EU | White | IS | M | lgsnd31867jq1 | EU | White | LGS | F |
| isnd31635di1 | EU | White | IS | M | lgsnd31894jz1 | EU | White | LGS | M |
| isnd31702dh1 | EU | White | IS | M | lgsnd31959kb1 | EU | White | LGS | M |
| isnd31770cy1 | America | Hispanic | IS | F | lgsnd31961jp1 | EU | White | LGS | M |
| isnd31821dn1 | EU | White | IS | M | lgsnd32224jb1 | E Asia | Other | LGS | M |
| isnd31831do1 | EU | White | IS | F | lgsnd32239kg1 | EU | White | LGS | M |
| isnd31899dq1 | EU | White | IS | F | lgsnd32265jt1 | EU | White | LGS | M |

^a^Ancestry determined by LASER/TRACE software, ^b^IS = Infantile Spasms, LGS = Lennox Gastaut Syndrome

*Ancestries: C/S Asia = Central/South Asia, E Asia = East Asia, EU = European, ME = ME

**Supplementary Table 1** Continued: Ancestry and Phenotype Information for Epi4k probands

| **proband ID** | **TRACE^a^ ancestry*** | **Reported Ancestry** | **IS/LGS^b^** | **Sex** | **proband ID** | **TRACE ancestry** | **Reported Ancestry** | **IS/LGS** | **Sex** |
| --- | --- | --- | --- | --- | --- | --- | --- | --- | --- |
| isnd32065dv1 | EU | Other | IS | M | lgsnd32289ju1 | EU | Other | LGS | M |
| isnd32121dj1 | EU | White | IS | F | lgsnd32340kk1 | EU | White | LGS | M |
| isnd32131ce1 | CS/E Asia | White | IS | F | lgsnd32497jr1 | EU | White | LGS | M |
| isnd32132dw1 | EU | White | IS | M | lgsnd32552jy1 | EU | White | LGS | M |
| isnd32241ec1 | EU | White | IS | F | lgsnd32562jx1 | EU | White | LGS | F |
| isnd32398dz1 | C/S Asia | Other | IS | F | lgsnd32630ka1 | ME | White | LGS | F |
| isnd32464eb1 | EU | White | IS | F | lgsnd32670kl1 | EU | White | LGS | M |
| isnd32641du1 | EU | White | IS | M | lgsnd32727kd1 | EU | White | LGS | F |
| isnd32671ef1 | EU | White | IS | M | lgsnd32763ke1 | EU | White | LGS | M |
| isnd32722cd1 | EU | White | IS | F | lgsnd32802kf1 | EU | White | LGS | F |
| isnd32757ee1 | EU | White | IS | M | lgsnd32879kj1 | EU | White | LGS | F |
| isnd33197eg1 | Africa | African American | IS | F | lgsnd32890jl1 | EU | White | LGS | M |
| isnd33296eh1 | EU | White | IS | M | lgsnd33014iu1 | Africa | African American | LGS | M |
| isnd33322el1 | E Asia | Asian | IS | M | lgsnd33064kh1 | EU | White | LGS | F |
| isnd33342ej1 | EU | White | IS | M | lgsnd33323kc1 | EU | White | LGS | M |
| isnd33520bn1 | Africa | African American | IS | F | lgsnd33346km1 | EU | White | LGS | M |
| isnd33651ed1 | EU | White | IS | M | lgsnd33590kn1 | C/S Asia | White | LGS | F |
| isnd34077er1 | EU | White | IS | F | lgsnd33706ko1 | EU | White | LGS | F |
| isnd34116ff1 | C/S Asia | White | IS | F | lgsnd33762kq1 | EU | Other | LGS | F |
| isnd34128ep1 | C/S Asia | White | IS | F | lgsnd34131kr1 | EU | White | LGS | F |
| isnd34144ew1 | America | Hispanic | IS | M | lgsnd34164ks1 | EU | White | LGS | F |
| isnd34170dt1 | EU | White | IS | M | lgsnd34306ki1 | EU | White | LGS | M |
| isnd34274ex1 | EU | White | IS | M | lgsnd34424kv1 | EU | White | LGS | M |
| isnd34304ey1 | EU | White | IS | F | lgsnd34500kx1 | EU | White | LGS | F |
| isnd34338ez1 | EU | White | IS | M | lgsnd34528kz1 | EU | White | LGS | F |
| isnd34401fc1 | EU | White | IS | F | lgsnd34593kt1 | EU | White | LGS | M |
| isnd34404fa1 | Africa | African American | IS | F | lgsnd34816ku1 | ME | Other | LGS | M |
| isnd34430aq1 | EU | White | IS | M | lgsnd35136ky1 | EU | White | LGS | M |
| isnd34548fb1 | Africa | African American | IS | M | lgsnd35495kp1 | EU | White | LGS | M |
| isnd34680fj1 | C/S Asia | Other | IS | F | lgsnd35817lb1 | EU | Other | LGS | M |
| isnd34750fe1 | EU | White | IS | F | lgsnd36163lc1 | EU | White | LGS | M |
| isnd34962fh1 | EU | White | IS | F | lgsnd36210jc1 | Africa | African American | LGS | M |
| isnd34968fg1 | EU | White | IS | F | lgsnd36440le1 | EU | White | LGS | M |
| isnd35054fi1 | EU | Other | IS | F | lgsnd36798lg1 | Africa | African American | LGS | F |

^a^Ancestry determined by LASER/TRACE software, ^b^IS = Infantile Spasms, LGS = Lennox Gastaut Syndrome

*Ancestries: C/S Asia = Central/South Asia, E Asia = East Asia, EU = European, ME = ME

**Supplementary Table 2. Epi4k and 1000 genomes CH and homozygous counts and p-values, all ancestries, 1%MAF, top 50**

| **Gene** | **TRID^a^** | **Epi4k 1% yes^b^** | **Epi4k 1% no** | **1kg^c^**  **1% yes** | **1kg 1% no** | **pval** | **Pval bh^d^** | **Pval bonf^e^** | **OR^f^** |
| --- | --- | --- | --- | --- | --- | --- | --- | --- | --- |
| PRTG | ENST00000389286 | 3 | 261 | 0 | 2504 | 0.000858684 | 1 | 1 | Inf |
| DNAJC4 | ENST00000321460 | 2 | 262 | 0 | 2504 | 0.009065347 | 1 | 1 | Inf |
| DNAJC4 | ENST00000321685 | 2 | 262 | 0 | 2504 | 0.009065347 | 1 | 1 | Inf |
| OSBP2 | ENST00000332585 | 2 | 262 | 0 | 2504 | 0.009065347 | 1 | 1 | Inf |
| OSBP2 | ENST00000382310 | 2 | 262 | 0 | 2504 | 0.009065347 | 1 | 1 | Inf |
| OSBP2 | ENST00000446658 | 2 | 262 | 0 | 2504 | 0.009065347 | 1 | 1 | Inf |
| MUC16 | ENST00000397910 | 11 | 253 | 215 | 2289 | 0.01244148 | 1 | 1 | 0.462993427 |
| FLG | ENST00000368799 | 4 | 260 | 113 | 2391 | 0.015655138 | 1 | 1 | 0.325614508 |
| TNC | ENST00000345230 | 3 | 261 | 4 | 2500 | 0.022454859 | 1 | 1 | 7.172904306 |
| TNC | ENST00000423613 | 3 | 261 | 4 | 2500 | 0.022454859 | 1 | 1 | 7.172904306 |
| TNC | ENST00000537320 | 3 | 261 | 4 | 2500 | 0.022454859 | 1 | 1 | 7.172904306 |
| C9orf114 | ENST00000361256 | 2 | 262 | 1 | 2503 | 0.025478672 | 1 | 1 | 19.0610251 |
| TNC | ENST00000346706 | 3 | 261 | 5 | 2499 | 0.033434418 | 1 | 1 | 5.737893801 |
| PKD1 | ENST00000262304 | 1 | 263 | 59 | 2445 | 0.040946041 | 1 | 1 | 0.15762227 |
| PKD1 | ENST00000423118 | 1 | 263 | 59 | 2445 | 0.040946041 | 1 | 1 | 0.15762227 |
| TNC | ENST00000340094 | 3 | 261 | 6 | 2498 | 0.04668945 | 1 | 1 | 4.780720266 |
| TNC | ENST00000341037 | 3 | 261 | 6 | 2498 | 0.04668945 | 1 | 1 | 4.780720266 |
| TNC | ENST00000350763 | 3 | 261 | 6 | 2498 | 0.04668945 | 1 | 1 | 4.780720266 |
| ANTXRL | ENST00000447511 | 3 | 261 | 6 | 2498 | 0.04668945 | 1 | 1 | 4.780720266 |
| TNC | ENST00000535648 | 3 | 261 | 6 | 2498 | 0.04668945 | 1 | 1 | 4.780720266 |
| TNC | ENST00000542877 | 3 | 261 | 6 | 2498 | 0.04668945 | 1 | 1 | 4.780720266 |
| ABCC11 | ENST00000353782 | 2 | 262 | 2 | 2502 | 0.047765771 | 1 | 1 | 9.533392929 |
| ABCC11 | ENST00000356608 | 2 | 262 | 2 | 2502 | 0.047765771 | 1 | 1 | 9.533392929 |
| ABCC11 | ENST00000394747 | 2 | 262 | 2 | 2502 | 0.047765771 | 1 | 1 | 9.533392929 |
| ABCC11 | ENST00000394748 | 2 | 262 | 2 | 2502 | 0.047765771 | 1 | 1 | 9.533392929 |
| PTCHD3 | ENST00000438700 | 2 | 262 | 2 | 2502 | 0.047765771 | 1 | 1 | 9.533392929 |
| MUC17 | ENST00000306151 | 1 | 263 | 51 | 2453 | 0.056611754 | 1 | 1 | 0.182941761 |
| NLRX1 | ENST00000409265 | 2 | 262 | 3 | 2501 | 0.074665107 | 1 | 1 | 6.355667514 |
| SCN10A | ENST00000449082 | 2 | 262 | 3 | 2501 | 0.074665107 | 1 | 1 | 6.355667514 |
| NLRX1 | ENST00000525863 | 2 | 262 | 3 | 2501 | 0.074665107 | 1 | 1 | 6.355667514 |
| FUK | ENST00000571514 | 2 | 262 | 3 | 2501 | 0.074665107 | 1 | 1 | 6.355667514 |
| MUC17 | ENST00000379439 | 1 | 263 | 48 | 2456 | 0.082911884 | 1 | 1 | 0.194612284 |
| AHNAK2 | ENST00000333244 | 3 | 261 | 80 | 2424 | 0.083759229 | 1 | 1 | 0.348364788 |
| CX3CL1 | ENST00000006053 | 1 | 263 | 0 | 2504 | 0.095375723 | 1 | 1 | Inf |
| TSPAN32 | ENST00000182290 | 1 | 263 | 0 | 2504 | 0.095375723 | 1 | 1 | Inf |
| CEACAM21 | ENST00000187608 | 1 | 263 | 0 | 2504 | 0.095375723 | 1 | 1 | Inf |
| C19orf26 | ENST00000215376 | 1 | 263 | 0 | 2504 | 0.095375723 | 1 | 1 | Inf |
| RNF215 | ENST00000215798 | 1 | 263 | 0 | 2504 | 0.095375723 | 1 | 1 | Inf |
| UBFD1 | ENST00000219638 | 1 | 263 | 0 | 2504 | 0.095375723 | 1 | 1 | Inf |
| KIAA1199 | ENST00000220244 | 1 | 263 | 0 | 2504 | 0.095375723 | 1 | 1 | Inf |
| TGFB1 | ENST00000221930 | 1 | 263 | 0 | 2504 | 0.095375723 | 1 | 1 | Inf |
| CRX | ENST00000221996 | 1 | 263 | 0 | 2504 | 0.095375723 | 1 | 1 | Inf |
| PRDM2 | ENST00000235372 | 1 | 263 | 0 | 2504 | 0.095375723 | 1 | 1 | Inf |
| NT5C1A | ENST00000235628 | 1 | 263 | 0 | 2504 | 0.095375723 | 1 | 1 | Inf |
| DHDSS | ENST00000236342 | 1 | 263 | 0 | 2504 | 0.095375723 | 1 | 1 | Inf |
| CAMSAP2 | ENST00000236925 | 1 | 263 | 0 | 2504 | 0.095375723 | 1 | 1 | Inf |
| CCDC92 | ENST00000238156 | 1 | 263 | 0 | 2504 | 0.095375723 | 1 | 1 | Inf |
| VIL1 | ENST00000248444 | 1 | 263 | 0 | 2504 | 0.095375723 | 1 | 1 | Inf |
| STRIP2 | ENST00000249344 | 1 | 263 | 0 | 2504 | 0.095375723 | 1 | 1 | Inf |

^a^TRID = Transcript ID, ^b^Count of subjects with CH or homozygous mutations, ^c^1kg = 1000 genomes, ^d^bh = Benamini Hochberg correction, ^e^bonf = Bonferroni correction, ^f^OR = Odds ratio

**Supplementary Table 3. Epi4k and 1000 genomes CH and homozygous counts and p-values, all ancestries, 0.5%MAF, top 50**

| **Gene** | **TRID^a^** | **Epi4k 0.5% yes^b^** | **Epi4k 0.5% no** | **1kg^c^**  **0.5% yes** | **1kg 1% no** | **pval** | **Pval bh^d^** | **Pval bonf^e^** | **OR^f^** |
| --- | --- | --- | --- | --- | --- | --- | --- | --- | --- |
| OSBP2 | ENST00000332585 | 2 | 262 | 0 | 2504 | 0.009065347 | 1 | 1 | Inf |
| OSBP2 | ENST00000382310 | 2 | 262 | 0 | 2504 | 0.009065347 | 1 | 1 | Inf |
| PRTG | ENST00000389286 | 2 | 262 | 0 | 2504 | 0.009065347 | 1 | 1 | Inf |
| OSBP2 | ENST00000446658 | 2 | 262 | 0 | 2504 | 0.009065347 | 1 | 1 | Inf |
| ABCC11 | ENST00000353782 | 2 | 262 | 1 | 2503 | 0.025478672 | 1 | 1 | 19.0610251 |
| ABCC11 | ENST00000356608 | 2 | 262 | 1 | 2503 | 0.025478672 | 1 | 1 | 19.0610251 |
| ABCC11 | ENST00000394747 | 2 | 262 | 1 | 2503 | 0.025478672 | 1 | 1 | 19.0610251 |
| ABCC11 | ENST00000394748 | 2 | 262 | 1 | 2503 | 0.025478672 | 1 | 1 | 19.0610251 |
| MACF1 | ENST00000289893 | 3 | 261 | 6 | 2498 | 0.04668945 | 1 | 1 | 4.780720266 |
| STAB1 | ENST00000321725 | 2 | 262 | 2 | 2502 | 0.047765771 | 1 | 1 | 9.533392929 |
| TNC | ENST00000345230 | 2 | 262 | 2 | 2502 | 0.047765771 | 1 | 1 | 9.533392929 |
| TNC | ENST00000423613 | 2 | 262 | 2 | 2502 | 0.047765771 | 1 | 1 | 9.533392929 |
| TNC | ENST00000537320 | 2 | 262 | 2 | 2502 | 0.047765771 | 1 | 1 | 9.533392929 |
| TNC | ENST00000340094 | 2 | 262 | 3 | 2501 | 0.074665107 | 1 | 1 | 6.355667514 |
| TNC | ENST00000341037 | 2 | 262 | 3 | 2501 | 0.074665107 | 1 | 1 | 6.355667514 |
| TNC | ENST00000346706 | 2 | 262 | 3 | 2501 | 0.074665107 | 1 | 1 | 6.355667514 |
| TNC | ENST00000350763 | 2 | 262 | 3 | 2501 | 0.074665107 | 1 | 1 | 6.355667514 |
| TNC | ENST00000535648 | 2 | 262 | 3 | 2501 | 0.074665107 | 1 | 1 | 6.355667514 |
| TNC | ENST00000542877 | 2 | 262 | 3 | 2501 | 0.074665107 | 1 | 1 | 6.355667514 |
| MACF1 | ENST00000372915 | 3 | 261 | 8 | 2496 | 0.079581706 | 1 | 1 | 3.583524267 |
| MACF1 | ENST00000564288 | 3 | 261 | 8 | 2496 | 0.079581706 | 1 | 1 | 3.583524267 |
| MACF1 | ENST00000567887 | 3 | 261 | 8 | 2496 | 0.079581706 | 1 | 1 | 3.583524267 |
| CX3CL1 | ENST00000006053 | 1 | 263 | 0 | 2504 | 0.095375723 | 1 | 1 | Inf |
| LLGL2 | ENST00000167462 | 1 | 263 | 0 | 2504 | 0.095375723 | 1 | 1 | Inf |
| CEACAM21 | ENST00000187608 | 1 | 263 | 0 | 2504 | 0.095375723 | 1 | 1 | Inf |
| UBFD1 | ENST00000219638 | 1 | 263 | 0 | 2504 | 0.095375723 | 1 | 1 | Inf |
| KIAA1199 | ENST00000220244 | 1 | 263 | 0 | 2504 | 0.095375723 | 1 | 1 | Inf |
| TFR2 | ENST00000223051 | 1 | 263 | 0 | 2504 | 0.095375723 | 1 | 1 | Inf |
| TIMELESS | ENST00000229201 | 1 | 263 | 0 | 2504 | 0.095375723 | 1 | 1 | Inf |
| PRDM2 | ENST00000235372 | 1 | 263 | 0 | 2504 | 0.095375723 | 1 | 1 | Inf |
| DHDDS | ENST00000236342 | 1 | 263 | 0 | 2504 | 0.095375723 | 1 | 1 | Inf |
| CCDC92 | ENST00000238156 | 1 | 263 | 0 | 2504 | 0.095375723 | 1 | 1 | Inf |
| AHDC1 | ENST00000247087 | 1 | 263 | 0 | 2504 | 0.095375723 | 1 | 1 | Inf |
| VIL1 | ENST00000248444 | 1 | 263 | 0 | 2504 | 0.095375723 | 1 | 1 | Inf |
| STRIP2 | ENST00000249344 | 1 | 263 | 0 | 2504 | 0.095375723 | 1 | 1 | Inf |
| E2F8 | ENST00000250024 | 1 | 263 | 0 | 2504 | 0.095375723 | 1 | 1 | Inf |
| CHRNA10 | ENST00000250699 | 1 | 263 | 0 | 2504 | 0.095375723 | 1 | 1 | Inf |
| KRT12 | ENST00000251643 | 1 | 263 | 0 | 2504 | 0.095375723 | 1 | 1 | Inf |
| LPIN1 | ENST00000256720 | 1 | 263 | 0 | 2504 | 0.095375723 | 1 | 1 | Inf |
| RALGAPA1 | ENST00000258840 | 1 | 263 | 0 | 2504 | 0.095375723 | 1 | 1 | Inf |
| ARHGAP20 | ENST00000260283 | 1 | 263 | 0 | 2504 | 0.095375723 | 1 | 1 | Inf |
| BCAR3 | ENST00000260502 | 1 | 263 | 0 | 2504 | 0.095375723 | 1 | 1 | Inf |
| KHK | ENST00000260599 | 1 | 263 | 0 | 2504 | 0.095375723 | 1 | 1 | Inf |
| ADCY3 | ENST00000260600 | 1 | 263 | 0 | 2504 | 0.095375723 | 1 | 1 | Inf |
| EXOC6 | ENST00000260762 | 1 | 263 | 0 | 2504 | 0.095375723 | 1 | 1 | Inf |
| PZP | ENST00000261336 | 1 | 263 | 0 | 2504 | 0.095375723 | 1 | 1 | Inf |
| TRPV4 | ENST00000261740 | 1 | 263 | 0 | 2504 | 0.095375723 | 1 | 1 | Inf |
| TELO2 | ENST00000262319 | 1 | 263 | 0 | 2504 | 0.095375723 | 1 | 1 | Inf |
| CCM2L | ENST00000262659 | 1 | 263 | 0 | 2504 | 0.095375723 | 1 | 1 | Inf |

^a^TRID = Transcript ID, ^b^Count of subjects with CH or homozygous mutations, ^c^1kg = 1000 genomes, ^d^bh = Benamini Hochberg correction, ^e^bonf = Bonferroni correction, ^f^OR = Odds ratio

**Supplementary Table 4. Epi4k and 1000 genomes CH and homozygous counts and p-values, EU ancestry, 1%MAF, top 50**

| **Gene** | **TRID^a^** | **Epi4k 1% yes^b^** | **Epi4k 1% no** | **1kg^c^**  **1% yes** | **1kg 1% no** | **pval** | **Pval bh^d^** | **Pval bonf^e^** | **OR^f^** |
| --- | --- | --- | --- | --- | --- | --- | --- | --- | --- |
| PRTG | ENST00000389286 | 3 | 204 | 0 | 503 | 0.024527517 | 1 | 1 | Inf |
| TTN | ENST00000342992 | 28 | 179 | 43 | 460 | 0.053560126 | 1 | 1 | 1.67202516 |
| OBSCN | ENST00000366707 | 4 | 203 | 2 | 501 | 0.063150347 | 1 | 1 | 4.923199244 |
| OBSCN | ENST00000422127 | 7 | 200 | 6 | 497 | 0.063251543 | 1 | 1 | 2.894171878 |
| MACF1 | ENST00000289893 | 3 | 204 | 1 | 502 | 0.076878328 | 1 | 1 | 7.358758844 |
| SYNE2 | ENST00000357395 | 3 | 204 | 1 | 502 | 0.076878328 | 1 | 1 | 7.358758844 |
| MACF1 | ENST00000372915 | 3 | 204 | 1 | 502 | 0.076878328 | 1 | 1 | 7.358758844 |
| SYNE2 | ENST00000394768 | 3 | 204 | 1 | 502 | 0.076878328 | 1 | 1 | 7.358758844 |
| MUC2 | ENST00000441003 | 3 | 204 | 1 | 502 | 0.076878328 | 1 | 1 | 7.358758844 |
| SYNE2 | ENST00000555002 | 3 | 204 | 1 | 502 | 0.076878328 | 1 | 1 | 7.358758844 |
| MACF1 | ENST00000564288 | 3 | 204 | 1 | 502 | 0.076878328 | 1 | 1 | 7.358758844 |
| MACF1 | ENST00000567887 | 3 | 204 | 1 | 502 | 0.076878328 | 1 | 1 | 7.358758844 |
| TNC | ENST00000340094 | 2 | 205 | 0 | 503 | 0.084709668 | 1 | 1 | Inf |
| TNC | ENST00000341037 | 2 | 205 | 0 | 503 | 0.084709668 | 1 | 1 | Inf |
| TNC | ENST00000345230 | 2 | 205 | 0 | 503 | 0.084709668 | 1 | 1 | Inf |
| TNC | ENST00000346706 | 2 | 205 | 0 | 503 | 0.084709668 | 1 | 1 | Inf |
| TNC | ENST00000350763 | 2 | 205 | 0 | 503 | 0.084709668 | 1 | 1 | Inf |
| ABCC11 | ENST00000353782 | 2 | 205 | 0 | 503 | 0.084709668 | 1 | 1 | Inf |
| ABCC11 | ENST00000356608 | 2 | 205 | 0 | 503 | 0.084709668 | 1 | 1 | Inf |
| SYNE1 | ENST00000356820 | 2 | 205 | 0 | 503 | 0.084709668 | 1 | 1 | Inf |
| C9orf114 | ENST00000361256 | 2 | 205 | 0 | 503 | 0.084709668 | 1 | 1 | Inf |
| ZZEF1 | ENST00000381638 | 2 | 205 | 0 | 503 | 0.084709668 | 1 | 1 | Inf |
| SDK2 | ENST00000388726 | 2 | 205 | 0 | 503 | 0.084709668 | 1 | 1 | Inf |
| SDK2 | ENST00000392650 | 2 | 205 | 0 | 503 | 0.084709668 | 1 | 1 | Inf |
| ABCC11 | ENST00000394747 | 2 | 205 | 0 | 503 | 0.084709668 | 1 | 1 | Inf |
| ABCC11 | ENST00000394748 | 2 | 205 | 0 | 503 | 0.084709668 | 1 | 1 | Inf |
| TNC | ENST00000423613 | 2 | 205 | 0 | 503 | 0.084709668 | 1 | 1 | Inf |
| OBSCN | ENST00000441106 | 2 | 205 | 0 | 503 | 0.084709668 | 1 | 1 | Inf |
| TNC | ENST00000535648 | 2 | 205 | 0 | 503 | 0.084709668 | 1 | 1 | Inf |
| TNC | ENST00000537320 | 2 | 205 | 0 | 503 | 0.084709668 | 1 | 1 | Inf |
| TNC | ENST00000542877 | 2 | 205 | 0 | 503 | 0.084709668 | 1 | 1 | Inf |
| TTN | ENST00000591111 | 28 | 179 | 46 | 457 | 0.104033319 | 1 | 1 | 1.553012662 |
| OBSCN | ENST00000570156 | 7 | 200 | 7 | 496 | 0.132113123 | 1 | 1 | 2.476401909 |
| TTN | ENST00000589042 | 28 | 179 | 50 | 453 | 0.186519554 | 1 | 1 | 1.41648123 |
| TTN | ENST00000342175 | 18 | 189 | 30 | 473 | 0.191266503 | 1 | 1 | 1.5006686 |
| TTN | ENST00000359218 | 18 | 189 | 30 | 473 | 0.191266503 | 1 | 1 | 1.5006686 |
| TTN | ENST00000460472 | 18 | 189 | 30 | 473 | 0.191266503 | 1 | 1 | 1.5006686 |
| MACF1 | ENST00000317713 | 2 | 205 | 1 | 502 | 0.205073971 | 1 | 1 | 4.884989822 |
| DNAH11 | ENST00000328843 | 2 | 205 | 1 | 502 | 0.205073971 | 1 | 1 | 4.884989822 |
| PCNT | ENST00000359568 | 2 | 205 | 1 | 502 | 0.205073971 | 1 | 1 | 4.884989822 |
| MACF1 | ENST00000361689 | 2 | 205 | 1 | 502 | 0.205073971 | 1 | 1 | 4.884989822 |
| MDN1 | ENST00000369393 | 2 | 205 | 1 | 502 | 0.205073971 | 1 | 1 | 4.884989822 |
| MACF1 | ENST00000372925 | 2 | 205 | 1 | 502 | 0.205073971 | 1 | 1 | 4.884989822 |
| DNAH11 | ENST00000409508 | 2 | 205 | 1 | 502 | 0.205073971 | 1 | 1 | 4.884989822 |
| MDN1 | ENST00000428876 | 2 | 205 | 1 | 502 | 0.205073971 | 1 | 1 | 4.884989822 |
| MACF1 | ENST00000539005 | 2 | 205 | 1 | 502 | 0.205073971 | 1 | 1 | 4.884989822 |
| MACF1 | ENST00000545844 | 2 | 205 | 1 | 502 | 0.205073971 | 1 | 1 | 4.884989822 |
| HIVEP2 | ENST00000012134 | 1 | 206 | 0 | 503 | 0.291549296 | 1 | 1 | Inf |
| TNS1 | ENST00000171887 | 1 | 206 | 0 | 503 | 0.291549296 | 1 | 1 | Inf |

^a^TRID = Transcript ID, ^b^Count of subjects with CH or homozygous mutations, ^c^1kg = 1000 genomes, ^d^bh = Benamini Hochberg correction, ^e^bonf = Bonferroni correction, ^f^OR = Odds ratio

**Supplementary Table 5. Epi4k and 1000 genomes CH and homozygous counts and p-values, EU ancestry, 0.5%MAF, top 50**

| **Gene** | **TRID^a^** | **Epi4k 0.5% yes^b^** | **Epi4k 0.5% no** | **1kg^c^**  **0.5% yes** | **1kg 1% no** | **pval** | **Pval bh^d^** | **Pval bonf^e^** | **OR^f^** |
| --- | --- | --- | --- | --- | --- | --- | --- | --- | --- |
| MACF1 | ENST00000289893 | 3 | 204 | 0 | 503 | 0.024527517 | 1 | 1 | Inf |
| MACF1 | ENST00000372915 | 3 | 204 | 0 | 503 | 0.024527517 | 1 | 1 | Inf |
| MACF1 | ENST00000564288 | 3 | 204 | 0 | 503 | 0.024527517 | 1 | 1 | Inf |
| MACF1 | ENST00000567887 | 3 | 204 | 0 | 503 | 0.024527517 | 1 | 1 | Inf |
| OBSCN | ENST00000366707 | 4 | 203 | 2 | 501 | 0.063150347 | 1 | 1 | 4.923199244 |
| MACF1 | ENST00000317713 | 2 | 205 | 0 | 503 | 0.084709668 | 1 | 1 | Inf |
| ABCC11 | ENST00000353782 | 2 | 205 | 0 | 503 | 0.084709668 | 1 | 1 | Inf |
| ABCC11 | ENST00000356608 | 2 | 205 | 0 | 503 | 0.084709668 | 1 | 1 | Inf |
| MACF1 | ENST00000361689 | 2 | 205 | 0 | 503 | 0.084709668 | 1 | 1 | Inf |
| MACF1 | ENST00000372925 | 2 | 205 | 0 | 503 | 0.084709668 | 1 | 1 | Inf |
| PRTG | ENST00000389286 | 2 | 205 | 0 | 503 | 0.084709668 | 1 | 1 | Inf |
| ABCC11 | ENST00000394747 | 2 | 205 | 0 | 503 | 0.084709668 | 1 | 1 | Inf |
| ABCC11 | ENST00000394748 | 2 | 205 | 0 | 503 | 0.084709668 | 1 | 1 | Inf |
| OBSCN | ENST00000441106 | 2 | 205 | 0 | 503 | 0.084709668 | 1 | 1 | Inf |
| MACF1 | ENST00000539005 | 2 | 205 | 0 | 503 | 0.084709668 | 1 | 1 | Inf |
| MACF1 | ENST00000545844 | 2 | 205 | 0 | 503 | 0.084709668 | 1 | 1 | Inf |
| MYO15A | ENST00000205890 | 2 | 205 | 1 | 502 | 0.205073971 | 1 | 1 | 4.884989822 |
| DNAH2 | ENST00000389173 | 2 | 205 | 1 | 502 | 0.205073971 | 1 | 1 | 4.884989822 |
| PKD1L2 | ENST00000525539 | 2 | 205 | 1 | 502 | 0.205073971 | 1 | 1 | 4.884989822 |
| PKD1L2 | ENST00000533478 | 2 | 205 | 1 | 502 | 0.205073971 | 1 | 1 | 4.884989822 |
| DNAH2 | ENST00000572933 | 2 | 205 | 1 | 502 | 0.205073971 | 1 | 1 | 4.884989822 |
| TTN | ENST00000342992 | 17 | 190 | 28 | 475 | 0.234492 | 1 | 1 | 1.516901915 |
| HIVEP2 | ENST00000012134 | 1 | 206 | 0 | 503 | 0.291549296 | 1 | 1 | Inf |
| TNS1 | ENST00000171887 | 1 | 206 | 0 | 503 | 0.291549296 | 1 | 1 | Inf |
| CEACAM21 | ENST00000187608 | 1 | 206 | 0 | 503 | 0.291549296 | 1 | 1 | Inf |
| TGM6 | ENST00000202625 | 1 | 206 | 0 | 503 | 0.291549296 | 1 | 1 | Inf |
| LAMB1 | ENST00000222399 | 1 | 206 | 0 | 503 | 0.291549296 | 1 | 1 | Inf |
| TFR2 | ENST00000223051 | 1 | 206 | 0 | 503 | 0.291549296 | 1 | 1 | Inf |
| DHDDS | ENST00000236342 | 1 | 206 | 0 | 503 | 0.291549296 | 1 | 1 | Inf |
| CCDC92 | ENST00000238156 | 1 | 206 | 0 | 503 | 0.291549296 | 1 | 1 | Inf |
| TICAM1 | ENST00000248244 | 1 | 206 | 0 | 503 | 0.291549296 | 1 | 1 | Inf |
| VIL1 | ENST00000248444 | 1 | 206 | 0 | 503 | 0.291549296 | 1 | 1 | Inf |
| E2F8 | ENST00000250024 | 1 | 206 | 0 | 503 | 0.291549296 | 1 | 1 | Inf |
| DHX29 | ENST00000251636 | 1 | 206 | 0 | 503 | 0.291549296 | 1 | 1 | Inf |
| KRT12 | ENST00000251643 | 1 | 206 | 0 | 503 | 0.291549296 | 1 | 1 | Inf |
| PLXNA1 | ENST00000251772 | 1 | 206 | 0 | 503 | 0.291549296 | 1 | 1 | Inf |
| AKAP12 | ENST00000253332 | 1 | 206 | 0 | 503 | 0.291549296 | 1 | 1 | Inf |
| DNHD1 | ENST00000254579 | 1 | 206 | 0 | 503 | 0.291549296 | 1 | 1 | Inf |
| LPIN1 | ENST00000256720 | 1 | 206 | 0 | 503 | 0.291549296 | 1 | 1 | Inf |
| RABEPK | ENST00000259460 | 1 | 206 | 0 | 503 | 0.291549296 | 1 | 1 | Inf |
| ARHGAP20 | ENST00000260283 | 1 | 206 | 0 | 503 | 0.291549296 | 1 | 1 | Inf |
| TRPV4 | ENST00000261740 | 1 | 206 | 0 | 503 | 0.291549296 | 1 | 1 | Inf |
| NUP153 | ENST00000262077 | 1 | 206 | 0 | 503 | 0.291549296 | 1 | 1 | Inf |
| FBN2 | ENST00000262464 | 1 | 206 | 0 | 503 | 0.291549296 | 1 | 1 | Inf |
| PDE4C | ENST00000262805 | 1 | 206 | 0 | 503 | 0.291549296 | 1 | 1 | Inf |
| KIF18A | ENST00000263181 | 1 | 206 | 0 | 503 | 0.291549296 | 1 | 1 | Inf |
| CLTCL1 | ENST00000263200 | 1 | 206 | 0 | 503 | 0.291549296 | 1 | 1 | Inf |
| LRP2 | ENST00000263816 | 1 | 206 | 0 | 503 | 0.291549296 | 1 | 1 | Inf |
| TECTA | ENST00000264037 | 1 | 206 | 0 | 503 | 0.291549296 | 1 | 1 | Inf |

^a^TRID = Transcript ID, ^b^Count of subjects with CH or homozygous mutations, ^c^1kg = 1000 genomes, ^d^bh = Benamini Hochberg correction, ^e^bonf = Bonferroni correction, ^f^OR = Odds ratio
